# COVID-19 and Vaccination: Knowledge, Attitudes and Practices of People Working on Illegal Gold Mining Sites in French Guiana

**DOI:** 10.3390/vaccines11071265

**Published:** 2023-07-21

**Authors:** Pierre Durand, Célia Basurko, Stephen Vreden, Mathieu Nacher, Maylis Douine

**Affiliations:** 1 Antilles Guyane Clinical Investigation Center, CIC Inserm 1424, Cayenne Hospital, Av des Flamboyants, 97300 Cayenne, France; celia.basurko@ch-cayenne.fr (C.B.); mathieu.nacher@ch-cayenne.fr (M.N.); 2 Internal Medicine and Infectious Diseases, Academic Hospital of Paramaribo, Flustratt, Paramaribo, Suriname; stephenvreden@yahoo.fr; 3 Foundation for scientific research (SWOS), Paramaribo, Suriname

**Keywords:** COVID-19, French Guiana, Brazil, Suriname, Amazonian Forest, illegal gold mining, undocumented migrants, vulnerable social group, self-medication, vaccination

## Abstract

Introduction: French Guiana is a French territory bordering Brazil and Suriname where the COVID-19 pandemic has severely strained the French Guianese health system. The people working on illegal gold mining sites in French Guiana, also known as garimpeiros, are mainly of Brazilian origin. Their health conditions are precarious, they live under the radar of the surveillance system and therefore, assessment of their health is quite challenging.. The objective of this study was to describe their knowledge, attitudes and practices regarding COVID-19 and vaccination against this infection. Methods: We conducted an international multicenter cross-sectional survey between 1 March 2022 and 30 April 2022 in French Guiana and Suriname, using a structured questionnaire. Results: Eighty persons were included, 95.0% of whom were Brazilian. Most had good general knowledge of COVID-19. Antibiotic prophylaxis had been practiced by 10.0% of participants. Forty-three people thought they had been infected with COVID-19 (53.8%). Self-medication was frequent, often with antibiotics (32.6%, mostlychloroquine, ivermectin or azithromycin) and most had not consulted a physician for symptoms of COVID-19. A majority (62.5%) had received at least one dose of vaccine. Those who were ever tested for COVID-19 were the most likely to be vaccinated (PR = 1.98, *p* = 0.009). Conclusions: Garimpeiros have a good level of knowledge about COVID-19 but the high consumption of antibiotics raises concerns about the selection of resistant bacteria. The vaccination rate was higher than that of the Guianese population. The most vaccinated individuals were those who had already taken a COVID test suggesting that these individuals had more exposure to the disease, were more health conscious, or had easier access to health centers.

## 1. Introduction

Located in the Amazon forest, bordering Suriname and Brazil, French Guiana (FG) is the only French territory in South America. It is a territory with a high gold content and gold mining is regulated [1]. However, many clandestine small-scale gold miners, also called “garimpeiros”, mainly of Brazilian origin, are installed in isolated camps in the forest. This mobile and poorly known population is estimated to be around 10,000 in FG [2,3]. The police repression of illegal gold mining aims to neutralize the logistical flows. In the midst of this struggle, the complexity of access to gold mining sites makes it difficult to provide medical care and prevention to the people who live there, and to study their health. In this context, studies have alerted to the health risk posed by illegal gold mining sites, due to their significant health precariousness, difficulties in accessing care, and the absence of appropriate care [4,5]. These risks are mainly infectious (malaria, leptospirosis, leishmaniasis, STIs, etc.) but also chronic illnesses such as high blood pressure, or musculoskeletal disorders [5,6,7,8]. Self-medication, misuse of antimicrobials and the risk of resistance have also been highlighted [5].

On the Maroni River, which marks the border with Suriname, the multicultural town of Maripasoula, relatively isolated from the rest of FG (access only by plane or boat), has a health center that is accessible free of charge to all people in the region. Many of the illegal gold mining sites in western FG have logistical rear bases in the villages just across from Maripasoula, on the Surinamese side of the Maroni River. These rear bases are small settlements (Antonio do Brinco and Ronaldo) composed of shops, restaurants and hotels that constitute logistical nodes where the garimpeiros come to spend time to buy goods, rest, spend their gold, associate, or sometimes get closer to health care structures [3]. This proximity allows some garimpeiros, while staying on these rear bases, to cross the river to come and consult for free at the health center of Maripasoula [9].

When the Sars-CoV2 (COVID-19) pandemic occurred in early 2020, FG initially appeared spared [10], but next experienced several important epidemic waves, against the backdrop of a tense sanitary situation, requiring prolonged restraint measures [11,12]. The management of the COVID-19 epidemic was not the same in FG and its neighboring country Brazil. Indeed, Brazil was particularly troubled by a strong politicization of science and medicine [13]. Brazilian health institutions encouraged and protocolized the prescription of drugs such as hydroxychloroquine, ivermectin and nitazoxanide (called “COVID Kit”) to ambulatory patients with COVID-19 without scientific justification [13,14,15]. The DETECTOCov-19 study carried out between August and October 2020 in Brazil showed that, among those diagnosed with COVID-19, 56.0% had taken drugs to treat the disease, usually combining different drugs from the “COVID Kit” [16]. In May 2021 in Brazil, one in four people reported taking drugs from the COVID Kit for prophylaxis or treatment of COVID-19, including self-medication [17]. 

Understanding knowledge, attitudes, and practices about COVID-19 and COVID-19 vaccination is very important to improve health messages and thus improve prevention and management. Throughout the world, the literature on this topic is very extensive and depends on culture and context [18,19,20]. Therefore, it is difficult to extrapolate results from one study to other specific populations. To date, no such study has been conducted on COVID-19 in illegal mine workers in FG. However, understanding the knowledge andpractices of the disease in this vulnerable population virtually deprived from access to regular health care, is valuable for health care decision makers. 

In the context of a difficult social situation and a rapidly evolving and hard-to-predict evolution of the epidemic, this study aimed to describe the knowledge, attitudes, and practices about COVID-19 and the anti-COVID vaccination of people who have worked on illegal gold mines in FG since the beginning of the pandemic.

## 2. Methods

### 2.1. Plan of the Study

This study was a cross-sectional, descriptive, transborder, multicenter KAP (Knowledge, Attitudes and Practices) study, with inclusions in French Guiana and Suriname. Data collection was prospective using standardized structured questionnaires (Supplementary Material).

### 2.2. Study Population

The study population consisted of adults who had worked for at least one month in one or more illegal gold mining sites in FG since March 2020, the date of the declaration of the pandemic in COVID-19 by the WHO. Exclusion criteria were minors, individuals who did not wish to participate in the study, and individuals with cognitive impairment who could not give informed consent.

Subjects were randomly recruited. A target of 80 to 100 participants was set for this study.

### 2.3. Inclusion Process

Inclusions took place from 1 March 2022 to 30 April 2022. Due to administrative and security aspects as well as logistical limitations, it was not possible to conduct the inclusions directly at the illegal gold mining sites. The 3 inclusion sites were: the health center of Maripasoula (FG), and the rear bases of Antonio Do Brinco (Suriname), and Ronaldo (Suriname). The questionnaires were filled out by a medical resident, assisted by a Portuguese-speaking translator. 

### 2.4. Questionnaire

The study was conducted using a standardized, anonimizedquestionnaire (no directly identifying data). It lasted between 30 and 45 min and included a maximum of 81 questions divided into 5 categories:

o Inclusion criteria: Age, date and time spent on illegal gold mining sites.
o Socio-demographic criteria: Sex, country of birth and language of the participant, type and location of work activity, medical history.
o Knowledge and perception of COVID-19
o Attitudes and practices toward COVID-19: Medications used. Number of COVID-19 tests performed. COVID-19 test results. Use of medical consultation. Symptoms presented. Sequelae.
o Attitudes about COVID vaccination: Reported vaccination rate. Type of vaccine received.

These were mainly closed-ended single or multiple choice questions based on the literature [21,22]. Anti-infectious agents such as chloroquine, ivermectin and azithromycin were grouped under the term “antibiotics” and their prophylactic use under the term “antibiotic prophylaxis”.

### 2.5. Ethical and Regulatory Aspects

Individual oral and written information was delivered face-to-face, in the participant’s own language, and non-opposition was collected. 

The project was registered with the Health Data Hub (N°F20220217195238) and the Commission Nationale de l’Informatique et des Libertés. The research protocol was approved by the ethical committee of Suriname (CMWO (Commissie voor Mensgebonden Wetenschappelijk Onderzoek) N°CMWO 16/23).

### 2.6. Statistical Analysis

Categorical, nominal, or ordinal categorical variables were presented as counts, percentages, and confidence intervals of percentages. Quantitative variables were presented as median and interquartile range. Bivariate analyses were performed using a Chi-square test^2^ and Poisson regression to identify/study the relationship between being vaccinated against COVID-19 and the sociodemographic and behavioral characteristics of the respondents using prevalence ratio (PR). Statistical analyses were performed using Excel and Stata12.0 (StataCorp, College Station, TX, USA). The alpha risk was 5%.

## 3. Results

Between 1 March 2022, and 30 April 2022, 80 individuals were included. The refusal rate was negligible.

### 3.1. Socio-Demographic Characteristics

The socio-demographic characteristics of the participants are presented in Table 1. Almost all participants were Brazilian (95.0%), mostly from the state of Maranhão (57.9%). Most of the participants were men with a M/F sex ratio of 1.67. The median age was 44 years (IIQ = 36–51). Most participants had worked at two to four different mining sites since the beginning of the pandemic. Of all the people interviewed, 78.8% reported no medical history but five people reported HIV infection (6.3%, 95% CI = [0.9–11.6]).

### 3.2. Knowledge about COVID-19

Regarding knowledge about COVID-19, 75.0% of the participants had first heard about it on television (Table 2). A small proportion of the participants (3.8%, 3/80) did not think that COVID-19 existed, and 92.2% (71/77) of the remaining 77 participants thought that the country of first appearance of the virus was China. Furthermore, 62.3% did not know the biological origin of the virus and 20.8% thought that the virus had accidentally escaped from a laboratory.

The majority of respondents (68.8%) believe that a symptom-free COVID-19 patient is no longer contagious. The modes of contamination reported were coughing and sneezing (droplets) for 83.75% of participants. Asked about useful preventive measures (open-ended question), participants mentioned covering their mouth and nose (71.3%), avoiding crowds of unknown people (61.3%) and disinfecting their environment (56.3%).

Almost all participants (93.8%) had friends, relatives, or colleagues affected by COVID-19 and most (51/80, 63.8%) reported having relatives who died of COVID-19 since the start of the pandemic.

### 3.3. Attitudes and Practices Related to COVID-19

The majority felt concerned or somewhat concerned by COVID-19 (51.3%). More than a quarter (26.3%) considered themselves at risk for a severe form of COVID-19. The garimpeiros considered COVID-19 was a danger and thought that the barrier measures were effective. The most reported symptoms attributed to the infection were: fever (93.8%), myalgia (93.8%), anosmia (93.8%), followed by dysgeusia (92.5%), and cough (92.5%).

Most of the respondents (62.5%) reported that little had changed in their activities since the start of the pandemic, while 26.3% had stopped working at the gold mining site, and 8.8% had started working there since the start of the pandemic (Table 2).

Eight respondents (10.0%) had taken antimicrobialdrugs as prophylaxis against COVID-19: seven had taken azithromycin, five ivermectin and two chloroquine. These drugs had been obtained in Suriname (50.0%), French Guiana (25.0%) or Brazil (25.0%) and had been used both on the gold mining sites (50.0%) and on the resting sites (50.0%). There was no predominant dosage or duration of treatment for any of these treatments. Several participants also mentioned drinking tea as a prophylactic treatment. 

Among the 43 people who reported being infected with COVID-19, the symptoms experienced were: fever (81.4%), rhinitis (81.4%) and myalgia (81.4%) followed by diarrhea (74.4%) and dysgeusia (74.4%). Among them, the majority said they had voluntarily isolated themselves (69.8%). A quarter had been infected a second time (27.9%), including five second infections confirmed by nasal test. A significant proportion reported suffering from sequelae (18.6%): asthenia, cough, myalgia, and pharyngeal pain. Twenty-three did not seek medical attention at the time of infection. Antibiotic treatment for curative purposes was reported in 43.5% of the 23 persons who did not consult a doctor and in 20.0% of the 20 who had received a medical consultation. 

### 3.4. Anti-COVID Vaccination

Almost all participants (96.3%) trusted the French health authorities completely or somewhat, compared to 60.0% for the Brazilian authorities and 32.5% for the Surinamese authorities. Regarding vaccination, almost all (91.3%) trusted the information provided by the public authorities and 86.3% of participants were “very favorable” to vaccination in general. Regarding the properties of the vaccines against COVID, 76.3% thought that the vaccines against COVID were effective and 75.0% thought that they were safe.

Fifty participants (62.5% (95%CI = [53.0–74.1]) reported being vaccinated (at least one dose of vaccine), of which 78.0% (39/50) had received at least one dose in French Guiana. Seven respondents had received at least one dose in Brazil (14.0%) and five in Suriname (10.0%). Almost all (96.0%) had received their injection in a hospital or health center. Most of them (29/50, 58%) had a history of COVID-19 infection. Fifteen (30.0%) had received a single dose, 30 (60.0%) had received two doses of COVID vaccine, and five (10.0%) had received three doses. Of the 15 individuals who received a single dose of COVID vaccine, 10 (66.7%) had a history of COVID-19 infection thus were fully vaccinated at the time of the study.

A quarter (26.0%) had received at least one dose of BNT162b mRNA (Pfizer^®^-BioNTech^®^, Mainz, Germany), 8.0% had received at least one dose of VAXZEVRIA (Astrazeneca^®^, Cambridge, United Knigdom), and 6.0% had received at least one dose of Coronavac (Sinovac Biotech^®^, Beijing, China). More than half (62.0%) did not know which vaccine they had received.

The 30 people (37.5%) who had not been vaccinated against COVID-19 explained it by the lack of safety of the vaccines (23.3%), difficulties to get vaccinated in their place of living (20.0%), difficulties to access vaccination centers (20.0%), not needing it (16.7%), and fearing the shots (10.0%). Only 6.7% did not think the COVID vaccines worked, and 6.7% mentioned lack of information about COVID vaccines.

### 3.5. Bivariate Analysis

After bivariate analysis (Table 3), women seemed to be more vaccinated than men without statistically significant difference (PR = 1.2, *p =* 0.283) and there was no difference in vaccination coverage between age categories. Those who considered themselves to be in poor health appeared to be more often vaccinated, as did those with chronic diseases (PR = 1.3, *p =* 0.18). People who have already taken a COVID test are the most likely to be vaccinated against COVID-19 (PR = 1.98, *p =* 0.009). 

## 4. Discussion

This is the first study to describe KAP among COVID-19 in people working on illegal gold mining sites in FG. We found that this population is fairly familiar with the symptoms and prevention method, has often been exposed personally or by their entourage to COVID, consumes a lot of antimicrobial agents as prophylaxis or treatment for COVID-19, and has an estimated vaccination coverage at the time of the study of 62.5%.

### 4.1. Impact of the Pandemic on Gold Miners

The population of garimpeiros was strongly affected by the pandemic. More than half (53.8%) reported having been infected at least once, and the number of participants with relatives who died of the disease was high (51/80, 63.8%). These mortality results should be interpreted with caution because in our study, information on the location of relatives was not collected.This high mortality may be related to the health vulnerability of this population, which has already been highlighted in other studies [4,5] but also with the high mortality rate due to COVID-19 observed in Brazil, one of the countries with the highest number of deaths related to this disease since the beginning of the pandemic [23].

The profile of the participants included was similar to that of the gold miners before the pandemic [2,3,5]. They were mainly Brazilians from poor and rural states of Brazil (Maranhao state) with low levels of education [3,5,9]. The pandemic did not change the mining activity for 62.5% of the respondents while 26.3% had stopped working at the gold mining site. Similar results were found in another study among gold miners in Suriname [24]. Yet many factors could have disrupted their activity, such as the closure of borders or the fear of coming in places of higher density and therefore with more circulation of COVID-19. Indeed the border between French Guiana and Brazil was reopened on 20 December 2021 after 22 months of closure, a few weeks before the start of this study [25]. 

Although this study did not investigate the impact of COVID-19 on the well-being of this population, COVID-19 showed negative psychological distress in different regions of the world [26].

### 4.2. A Population with a Good Level of Knowledge about the COVID-19

In this study, the gold miners had good general knowledge about COVID-19 and seemed to understand the seriousness of this disease. However, their information network was limited and mainly consisted of television. This almost exclusively Portuguese-speaking population, was informed by the Brazilian media television channels, which corresponds to the observations made on the gold mining sites [3]. The knowledge of the participants on the country of occurrence of the virus, the modes of transmission, the protective measures, the risk factors and the symptoms was good. The biological mode of occurrence of the virus was less well known. Fake news did not seem to have a serious impact on the participants’ answers in this survey. The comparison with literature data is complicated due to great disparity of KAP between different populations and its great evolution over time. Furthermore, behaviour is not always linked to knowledge: adequate knowledge does not necessarily lead to an adequate attitude [19].

### 4.3. Attitudes and Practices Similar to Those Observed in the Brazilian Population

It is interesting to see that the gold miners, mainly from Brazil, have attitudes and practices more comparable to those of the population of Brazil than to those of French Guiana, where they live. Thus, the practice of anti-COVID antibiotic prophylaxis was comparable to that was observed in Brazilian studies. In the Datafolha study, 12.0% had taken medication for prevention, including antibiotics and other medications [17]. In the Detectcov study, 24.6% had taken preventive self-medication including 35.8% paracetamol, 35.8% ivermectin, 21.5% azithromycin, and only 1.3% hydroxychloroquine [16]. In our study, almost one third (32.6%) of the people had taken antimicrobial medication (including ivermectin) for curative purposes. Before antibiotics, the most frequently found drugs were vitamins and paracetamol, which corresponds to the most commonly used drugs for self-medication among gold miners [9]. Access to antibiotics is easy for gold miners as they are freely available on Suriname’s resting sites, in the informal sector called “Brazilian pharmacies” and from “Chinese” traders. These antibiotics, purchased and consumed in this way, can be poorly preserved or misused and are at risk of drug interactions, adverse effects and microbial resistance.

Preventive measures are little used: half of the garimpeiros declared respecting social distancing (51.3%) and hand disinfection (55.0%), even if they considered them “very effective”. These figures do not reflect the reality observed in the field and are probably influenced by the fact that the interviewer had a medical background. They can also be explained by the lifestyle of the garimpeiros, who lived and worked essentially in an open environment where the risk of contamination is lower. In addition, it is not always easy for them to consult health centers or even to isolate themselves when they have symptoms. The self-medication, including antibiotics, observed in the Brazilian population, appears therefore, in the eyes of the garimpeiros, as a quick and easy solution and is generally considered as effective.

### 4.4. Results to Be Considered in a Temporal Context

At the time of the development of the research project between December 2021 and January 2022, French Guiana was experiencing a new epidemic wave of COVID-19 linked to the SaRS-CoV2 then remain stable during the inclusion period [27]. At the time of inclusion, Suriname and Brazil were still classified as “orange” countries by the French Ministry of Interior, corresponding to active virus circulation.

Numerous studies have shown that KAP toward COVID-19 changed throughout the pandemic, depending on changes in medical knowledge, incidence rate, mortality rate, or perception of the policy response [28,29]. In this study, COVID-19 was considered severe and dangerous but the concern was moderate, probably related to the rather favorable epidemic situation at the time of our inclusions and the lower case fatality. Only repeated studies would allow to evaluate the trends of this perception and its relation to incidence.

### 4.5. Vaccination Rate Higher than that of the French Guianese Population

Nearly two out of three (62.5%) people in this study, had received at least one dose of COVID vaccine. This vaccination coverage appeared suprisingly high for a vulnerable and remote population. It was better than that of the general French Guianese population (45.0% of the eligible Guianese population) and closer to the Brazilian vaccination coverage (80.0%) at the time of the study [27,30]. This difference with the overall French Guianese population is all the more striking because western FG, where the commune of Maripasoula is located, had poorer vaccination coverage (7.0% in in August 2021 [31].

In our study, the main reason for not getting vaccinated was difficult access to the vaccine, followed by lack of vaccine safety. Indeed, illegal gold mines are located in remote areas of the forest, which can be up to 5 days by boat or walk from health centers. This is very different from the French Guianese population who was very suspicious about the safety of the vaccine and did not trust the health authorities and massively refused the vaccine [22,32]. Several studies have shown that vaccination seemed to be correlated with the level of concern and the level of education [22,32]. We found similar results, as participants with at least one previous COVID test were more likely to be vaccinated. These may be people with more exposure to the disease, with greater concern for their health, or with better knowledge of and access to health care facilities. A large majority of those vaccinated had received at least one dose in French Guiana. This might reflect a good level of trust in the French authorities, despite their illegal status, which would be interesting to study.

### 4.6. Strengths and Limitations

The strong point of this first study to focus on COVID-19 among gold miners was that it allowed for a multicentric collection of data, both in a health care facility and on the resting sites of the gold miners, and that it was carried out, in part, with a Portuguese-speaking Brazilian mediator who was familiar with the target population.

The main bias of our study was recruitment bias. Indeed, our inclusions were mainly on the resting sites of the gold miners, which are places of passage, with a probable over-representation of people who move around the most, who may have the most means, or who are the least afraid of moving around and mingling with the crowds of the resting sites. Since poor access to the vaccine is the main cause of non-vaccination, vaccination coverage in our study may be overestimated since we included more people moving around. In addition, those most concerned about their health may have participated more. Thus our snow-ball based sample may not be representative of the whole gold miningpopulation. The interviewer being from the medical field, the participants may have given the expected answers rather than their real knowledge and practices. The small sample size reduces the power of the study and therefore the possibility of highlighting differences in behavior or knowledge. These biases limit the representativeness of our study and thus its external validity.

## 5. Conclusions

Despite their remoteness and vulnerability, people working in FG’s illegal gold mines appear to have good knowledge of COVID-19 and are fairly well vaccinated. Given that these individuals are under the radar of the surveillance system, it was important to document KAP and vaccination coverage in this specific population. These data are of interest to healthcare institutions to better target this population and improve their health status. Improving health messages (especially limiting self-medication with antibiotics) and vaccination coverage could be done in partnership with Suriname, where the logistical rear bases of the gold mines are located, and through health mediators who are very important in empowering this community.

## Figures and Tables

**Table 1:** Socio-demographic characteristics of participants (*n* = 80).

		*n*	%
Age (years)			
	18–34	15	18.8
	35–49	36	45.0
	50–64	25	31.3
	65+	4	5.0
Place of recruitment			
	Maripasoula health center	28	35.0
	Antonio Do Brinco	28	35.0
	Ronaldo	24	30.0
Nationality			
	Brazil	76	95.0
	Other	4	5.0
Brazilian birth state			
	Maranhão	44	57.9
	Para	15	19.7
	Amapa	3	4.0
	Other	14	18.4
Level of education			
	Incomplete primary school or not enrolled	48	60.0
	Basic education cycle 1 complete	14	17.5
	Basic education cycle 2 completed	2	2.5
	High school with baccalaureate (vestibular)	15	18.8
	Graduate	1	1.3
Countries worked since March 2020 - outside France			
	Brazil	5	6.3
	Suriname	28	35.0
Countries visited since March 2020 - outside France			
	Brazil	24	30.0
	Suriname	76	95.0
	French Guiana	1	1.3
Number of sites since March 2020			
	1	20	25.0
	2–>4	43	53.8
	5–>9	14	17.5
	10+	3	3.8
Personal Health Assessment			
	Excellent	5	6.3
	Very good	13	16.3
	Good	20	25.0
	Reasonable	35	43.8
	Bad	7	8.8
Personal History			
	Diabetes	4	5.0
	HTA	11	13.8
	HIV	5	6.3
	No	63	78.8
Taking a long-term treatment			
Yes		29	36.3
No		51	63.8

**Table 2:** Knowledge, attitudes, and practices of the garimpeiros on the COVID-19 (March–April 2022).

		Number of Responses	*n*	%
Knowledge and Representations				
Risk factors for severe forms		80		
	Comorbidities		55	68.8
	Seniors		49	61.3
	Children/Babies		19	23.8
	Smoking		5	6.3
	Alcohol and tobacco		2	2.5
	Pregnancy		5	6.3
	Unvaccinated person		1	1.3
Perceived effectiveness of handwashing		80		
	Extremely efficient		7	8.8
	Very efficient		51	63.8
	Moderately effective		11	13.8
	Not very efficient		4	5.0
	Not effective		7	8.8
Perceived effectiveness of face mask use		80		
	Extremely efficient		5	6.3
	Very efficient		54	67.5
	Moderately effective		11	13.8
	Not very efficient		3	3.8
	Not effective		7	8.8
Will the COVID-19 disappear?		80		
	Yes, of course		28	35.0
	Yes, probably		12	15.0
	Not sure		11	13.8
	Probably not		8	10.0
	Certainly not		21	26.3
Perception of the dangerousness of COVID-19		80		
	A little dangerous		4	5.0
	Moderately dangerous		7	8.8
	Very dangerous		63	78.8
	Extremely dangerous		6	7.5
Perception of the threat of COVID-19 for humanity		80		
	Not serious		1	1.3
	Serious		15	18.8
	Very serious		62	77.5
	Don’t know		2	2.5
Concern about COVID-19		80		
	Not at all worried		11	13.8
	Not really worried		28	35.0
	Somewhat concerned		10	12.5
	Concerned		31	38.8
Attitudes and Practices				
Decrease in travel since the pandemic		80		
	Yes, due to restrictions and controls		18	22.5
	Yes, for fear of getting sick		28	35.0
	Yes, for fear of spreading the virus		5	6.3
	No, I had planned to travel and I stuck to my plans		14	17.5
	No, I wasn’t planning to travel.		24	30.0
	Other (Not due to financial reasons related to COVID-19)		1	1.3
Respect for social distance		80		
	Never/rarely		18	22.5
	Sometimes		21	26.3
	Often/always		41	51.3
Frequency of hand sanitization		80		
	Never/rarely		12	15.0
	Sometimes		14	17.5
	Often/always		44	55.0
Frequency of mask use at work		80		
	Never/rarely		58	72.5
	Sometimes		15	18.8
	Often/always		7	8.8
Has already done a COVID-19 test		80		
	Yes		63	78.3
	No		17	21.3
Where?		80		
	Yes in health center		56	70.0
	Yes, in a city/village screening campaign		9	11.3
	Yes, in garimpo		1	1.3
	Yes, Paramaribo laboratory		1	1.3
	Yes, pharmacy in Brazil		2	2.5
	No		17	21.3
If so, for what reason?		63		
	Symptoms		46	73.0
	Contact with patient		4	6.4
	Asymptomatic screening		20	31.8
Number of “COVID tests” performed since the beginning of the pandemic		63		
	1 time		21	33.3
	2 to 5 times		40	63.5
	6 to 10 times		2	3.2
Think you’ve had the COVID-19 since the start of the pandemic?		80		
	Yes		43	53.8
	No		30	37.5
	Don’t know		7	8.8
Has this been confirmed?		43		
	Yes, positive nasal test		24	55.8
	Yes, positive blood test		1	2.3
	No, just symptoms		37	86.1
Year of infection		43		
	2020		16	20.0
	2021		25	31.3
	2022		4	5.0
On the gold mining site during the infection?		43		
	Yes		22	51.2
	No		21	48.8
Country of infection		43		
	Brazil		8	18.6
	Suriname		16	37.2
	French Guiana		19	44.2
Medical consultation during the infection		43		
	Yes		20	46.5
	No		23	53.5
Type of consultation		20		
	Health Center		17	85.0
	Private physician		3	15.0
	Hospitalization		0	0.0
Taking medication to treat the infection?		43		
	Yes		37	86.1
	No		6	14.0
Taking antibiotics for curative purposes?		43		
	Yes		14	32.6
	No		29	67.4
Drugs used		37		
	Ivermectin		10	27.0
	Chloroquine		7	18.9
	Azithromycin		12	32.4
	Vitamins		15	40.5
	Paracetamol		26	70.3
Perception of the effectiveness of the treatment taken		37		
	Extremely efficient		1	2.7
	Very efficient		28	70.3
	Moderately effective		6	21.6
	Not very effective		2	5.4
Discussion with family and friends about COVID-19		80		
	Never/rarely		23	28.8
	Sometimes		27	33.8
	Often/all the time		30	37.5

**Table 3:** Sociodemographic and behavioral characteristics of vaccinated versus unvaccinated participants (CAPcovid19 2022, *n* = 80).

	Vaccinated (*n* = 50)	Not Vaccinated (*n* = 30)	*p*	PR * (95% CI)
	*n* (%)	*n* (%)	(Chi2)	(Poisson)
Being a woman	21/50 (42.0)	9/30 (30.0)	0.283	1.2 [0.69–2.12]
Be 44 years of age or older	25/50 (50.0)	16/30 (53.3)	0.773	0.95 [0.55–1.66]
Place of recruitment				
Maripasoula health center	19/50 (38.0)	9/30 (30.0)	0.756	1
Antonio Do Brinco	17/50 (34.0)	11/30 (36.7)		0.89 [0.47–1.72]
Ronaldo	14/50 (28.0)	10/30 (33.3)		0.86 [0.43–1.71]
Completed elementary school or higher	22/50 (44.0)	10/30 (33.0)	0.346	1.18 [0.67–2.06]
Work in more than 3 sites since	25/50 (50.0)	13/30 (43.3)	0.563	1.11 [0.63–1.92]
the beginning of pandemic				
Health personal assessment				
Good to very good	22/50 (44.0)	16/30 (53.3)		1
Poor to reasonable	28/50 (56.0)	14/30 (46.6)	0.418	1.15 [0.66–2.01]
Suffering from a chronic disease	13/50 (26.0)	4/30 (13.32)	0.18	1.3 [0.69–2.45]
(Diabetes, HTA, HIV, Cancer)				
Taking a long-term treatment	20/50 (40.0)	9/30 (30.0)	0.368	1.17 [0.67–2.06]
Thinking that COVID-19 will disappear	24/50 (48.0)	16/30 (53.3)	0.644	0.92 [0.53–1.61]
Feeling somewhat concerned/concerned	23/50 (46.0)	18/30 (59.9)	0.225	0.81 [0.46–1.41]
about COVID-19				
Having already done a COVID-19 test	44/50 (88.0)	19/30 (63.3)	0.009	1.98 [0.84–4.64]
Thinking to have already been infected	29/50 (58.0)	14/30 (46.6)	0.325	1.19 [0.68–2.08]
with COVID-19				
Having used antibiotics for curative purposes	11/50 (22.0)	3/30 (10.0)	0.279	1.27 [0.60–2.68]
Having relatives affected with COVID-19	47/50 (94.0)	28/30 (93.2)	905	1.04 [0.33–3.36]
Having relatives died from COVID-19	13/50 (26.0)	11/30 (36.6)	0.296	1.23 [0.65–2.33]
Frequency of discussions about COVID-19				
Never/rarely	14/50 (28.0)	9/30(10.0)	1	1
Sometimes/many	36/50(72.0)	21/30(69.9)	0.848	1.04 [0.56–1.92]

* PR: prevalence ratio.

## Data Availability

All data relevant to our analyses are fully described in the article and can be made available upon request. All data used for the analysis are available upon request from the corresponding author.

## Supplementary Materials

The following supporting information can be downloaded at: https://www.mdpi.com/article/10.3390/vaccines11071265/s1, Questionnaire.
