# Supplementary material for: COVID-19 and Vaccination: Knowledge, Attitudes and Practices of People Working on Illegal Gold Mining Sites in French Guiana
Source: Vaccines (Basel). 2023 Jul 21;11(7):1265. doi: 10.3390/vaccines11071265 (PMC10383914; doi:10.3390/vaccines11071265)
Supplement: Supplementary file 1 [file vaccines-11-01265-s001.zip › vaccines-2387678-supplementary.pdf]

CPF Knowledges, attitudes and practices about Covid-19 of people working on illegal gold mining sites in French Guiana.

A - Inclusion criteria

1 - Have you worked in an illegal gold mining site in French Guiana since March 2020 (Beginning of the sanitary lockdown in French Guiana and Brazil and closure of Guianese borders)? (Only one answer)
☐ Yes ☐No

2 - Did you work on this site for more than 1 month from March 2020? (Only one answer)
☐Yes ☐No

3 - How old are you? ____

4 - Do you agree to participate in this research conducted by Cayenne Hospital (French Guiana) (One answer only)
☐Yes ☐No

5 - Is this the first time you are participating in this study? (Only one answer)
☐Yes ☐No

Collection of informed consent? (Only one answer)
☐Yes ☐No

Patient Inclusion (One Response Only)
☐Yes ☐No

If yes :
Date of inclusion : _____/____/_________
Name of the person performing the inclusion:
____

Patient anonymity number :
____

Place of recruitment : (Only one answer)
☐CDPS of Maripasoula ☐City of Maripasoula ☐Antonio Do Brinco ☐Ronaldo
☐Other: ____

B - Socio-demographic criteria

6 -You are : (Only one answer)
☐A man ☐A woman
☐Other: ____

7 – Where were you born ? (Only one answer)
☐ Brazil ☐ Suriname ☐ French Guiana ☐ Guyana ☐ Venezuela ☐ France
☐ Another South American country
☐ Other: ____

7 - A - If Brazil, specify region: (One answer only)
☐Amapa ☐Para ☐Roraima ☐Maranhão
☐Autre :____

8 - What is the highest level of education you have achieved? (Only one answer)
☐Incomplete primary education or no schooling
☐Primary school completed
☐Secondary school
☐Higher education
☐Higher education degree / Post-graduation

9 - In which region(s) have you been mining for gold since March 2020?
(One or more answers)
☐Brazil ☐French Guiana ☐Suriname ☐Guyana ☐Venezuela
☐Other: ____

10 - In how many different gold mining sites have you worked since March 2020?
(free answer)

____

11 - In which region(s) have you travelled since Mars 2020? (One or more answers)
☐Brazil ☐French Guiana ☐Suriname ☐Guyana ☐Venezuela
☐Other: ____

12 - What job are you currently doing? (Only one answer)
☐I work on a gold mining site
☐I do another job
☐ I don't do anything
☐Other: ____

13 - How would you rate your overall health? (Only one answer)
☐Excellent ☐Very good ☐Good ☐Reasonable ☐Poor

14 - Do you have one of this medical history? (One or more answers)
☐Diabetes ☐Hypertension ☐HIV ☐Cancer
☐No

15 - Do you take medication every day for an orther disease than Covid-19? (Only one answer)
☐Yes ☐No

C - Knowledge and perceptions of Covid-19

16 - How did you hear about Covid-19 the first time? (Only one answer)
☐Radio ☐Television ☐Social networks ☐Print media
☐Word of mouth ☐Doctor/health professional
☐Other: ____
☐I don't know / I don't remember
☐I have never heard about Covid-19

17 - Do you think Covid-19 really exists? (Only one answer)
☐Yes ☐No

17 - A - If yes, in which country did the coronavirus (Covid-19) appear? (Only one answer)
☐France ☐Brazil ☐China ☐United States ☐Portugal
☐Other:
____

17 - B - If yes, in your opinion, what is the origin of the Covid-19? (Open question, one or more answer)
☐ Transmission by contact with an animal
☐ Accidental leak from a laboratory.
☐Propagated by the military to decrease the world population
☐Created by laboratories to sell vaccines.
☐I don't know.
☐Other:

18 - According to you, what are the symptoms of Covid-19? (One or more answers)
☐Fever ☐Cough ☐Lack of air ☐Sore throat ☐Runny or stuffy nose
☐Muscle or body aches ☐Headaches ☐Tiredness ☐Diarrhea
☐Loss of taste ☐Loss of smell
☐Other: ____
☐No symptoms

19 - In your opinion, does a person who has no symptoms of coronavirus can still be contagious? (Only one answer)
☐Yes ☐No

20 - : Which of the following best describes the mode of propagation of Covid-19? (Open question, one or more answer)
☐Human contact, coughing or sneezing ☐Pet contact
☐Consumption of game meat ☐Mosquito bites ☐Dirt or pollution
☐Sexual relationship ☐Air
☐Other: ___

21 - Do you think you have risk factors for a severe form of Covid-19? (Only one answer)
☐Yes ☐No
☐Don't know

21 - A - If yes, which ones? (Free text) ___
___

22 - Which of the following people are at risk of developing a severe form of Covid-19? (Open question, one or more answer)
☐People of certain religions
☐The elderly
☐Babies and young children
☐People already suffering from certain diseases
☐People with a certain ethnic background
☐None of the above
☐Other: ___

23 - How effective is hand washing in preventing the spread of Covid-19? (One answer only)
☐Extremely efficient ☐Very effective ☐Moderately effective
☐Not very effective ☐Not effective

24 - How effective is the use of a face mask in preventing the spread of Covid-19? (Only one answer)
☐Extremely efficient ☐Very effective ☐Moderately effective
☐Not very effective ☐Not effective

25 - What measures are effective in preventing the spread of Covid-19? (Open question, one or more answer)
☐Cover your mouth and nose when you cough or sneeze
☐Avoid close contact with people who have a fever or cough
☐Stay more than three feet away from other people when you are in public.
☐Avoiding crowds of unknown people
☐Stay on the gold mining sites and avoid returning to town.

☐Avoid touching eyes, nose and mouth with unwashed hands.
☐Take herbal supplements, tea and traditional remedies.
☐Taking antibiotics (chloroquine, Ivermectin, azithromycin..) or other medications.
☐Taking vitamins
☐Eating garlic, ginger or lemon
☐Drinking alcoholic beverages
☐Smoking tobacco
☐Be careful when opening letters or packages
☐Be vaccinated against flu/Influenza
☐Cleaning or disinfecting your environment
☐Cleaning or disinfecting your mobile phone
☐Other: ____
☐None of the options

26 - Do you think that Covid-19 will disappear one day? (Only one answer)
☐Yes, of course
☐Yes, probably.
☐Not on
☐Probably not
☐Certainly not

27 - How dangerous do you think Covid-19 is for your community? (Only one answer)
☐Nothing dangerous ☐Somewhat dangerous ☐Moderately dangerous
☐Very dangerous ☐Extremely dangerous

28 - How do you assess the level of seriousness of the Coronavirus (Covid-19) disease for humanity? (Only one answer)
☐Not serious ☐Serious ☐Very serious ☐Don't know

29 - How do you feel about the coronavirus pandemic (Covid-19)? (Only one answer)
☐Not at all concerned ☐Somewhat not concerned ☐Somewhat concerned ☐ Very concerned

D - Attitudes and practices towards Covid-19

30 - How has your work changed since the beginning of the Covid-19 pandemic? (One answer only)
☐Not much has changed
☐I started working on the gold mining sites
☐I stopped working at the gold mining sites
☐I had to change my gold mining site.

31 - Have you cancelled or reduced your travel since the start of the Covid-19 pandemic? (One or more answers)
☐Yes, due to restrictions and controls
☐Yes, for fear of getting sick
☐Yes, for fear of spreading the virus
☐No, I had planned to travel and I stuck to my plans
☐No, I wasn't planning to travel.
☐Other: ____

32 - How often do you respect social distanciating ?(Only one answer)
☐Never ☐Rarely ☐Sometimes ☐Often ☐Always

33 - How often do you sanitize your hands with soap or hydroalcoholic solution? (Only one answer)
☐Never ☐Rarely ☐Sometimes ☐Often ☐Always

34 - How often do you wear a face mask when you are with people? (Only one answer)
☐Never ☐Rarely ☐Sometimes ☐Often ☐Always

35 - Did you take any medication to protect yourself from Covid-19? (One or more answers)
☐Yes, when I was on a gold mining site
☐Yeah, when I was in town
☐No

35 - A - If yes, which ones? (One or more answers)
☐Ivermectin ☐Chloroquine ☐Azithromycin ☐Vitamins :
☐Yes but I don't know the name.
☐Yes, other :
____

35 - A1 - If Chloroquine, how often and for how long did you take chloroquine? (Only one answer)
☐Once a month ☐Once a week ☐Once a day
☐Only when in contact with sick people.
☐Other: ____
How long? (free text) ____________________________________

35 - A2 - If Ivermectin, how often and for how long did you take Ivermectin? (Only one answer)
☐Once a month ☐Once a week ☐Once a day
☐Only when in contact with sick people.
☐Other: ____
How long? (free text) ____________________________________

35 - A3 - If Azithromycin, how often and for how long did you take azithromycin? (Only one answer)
☐Once a month ☐Once a week ☐Once a day
☐Only when in contact with sick people.
☐Other: ____
How long? (free text) _______________________________________

35 - B - If yes, where did you get these medicines ? (One or more answers)
☐Brazil ☐Guyana ☐Suriname ☐French Guiana
☐Other: ____

35 - C - If yes, how did you get these medicines ? (One or more answers)
☐Commercial Pharmacy
☐Other shops in a village or town
☐Seller on the gold mining site
☐Health dispensary or clinic
☐Hospital
☐Other: ____

36 - Have you ever made a Covid-19 test? (One or more answers)
☐Yes, in a hospital or health center
☐Yes, during a detection campaign in a town or village
☐Yes in a garimpo (by my self or someone else)
☐Other: ____
☐No

36 - A - If yes, for what reason(s)? (One or more answers)
☐Because I had symptoms
☐Because I was in contact with sick people
☐In detection when I was asymptomatic
☐I don't know/remember

36 - B - If yes, how many times have you done this since the beginning of the pandemic in March 2020? (Only one answer)
☐Only once ☐2 to 5 times ☐6 to 10 times ☐>10 times

37 - Do you think you have been sick from Covid-19 since the beginning of the pandemic in March 2020? (Only one answer)
☐Yes, I did a nasal test and it was positive
☐Yes, I did a blood test and it was positive
☐Yes, I had the symptoms of Covid-19
☐No
☐I don't know/I can't remember

37- A – If yes, when was it ? (Open question)

____
______

37 - B - If yes, what were the symptoms you felt? (One or more answers)
☐Fever ☐Cough ☐Lack of air ☐Sore throat
☐Muscle or body aches ☐Headaches ☐Tiredness ☐Diarrhea
☐Loss of taste ☐Loss of sense of smell ☐Runny or stuffy nose
☐Other: ____
☐No symptoms

37 - C - If yes, were you in a gold mining site at that time? (Only one answer)
☐Yes ☐No
☐I don't know, I can't remember

37 - D - If yes, in which country were you at that time? (Only one answer)
☐ Brazil ☐ Suriname ☐ French Guiana ☐ Guyana ☐ Venezuela
☐ Another South American country
☐ Other: ____

37 - E - If yes, did you consult or were you followed by health professionals during your Covid-19? (One or more answers)
☐No
☐Yes, in a health center in a town or village
☐Yes, a private doctor
☐Yes, I was hospitalized

37 - F - If yes, did you take medication to cure this illness? (One or more answers)
☐Ivermectin ☐Chloroquine ☐Azithromycin ☐Vitamins :
☐Yes, other : ____
☐Yes but I don't know the name.
☐No

37 - F1 - If yes, do you think those medicines have been effective? (Only one answer)
☐Extremely efficient ☐Very effective ☐Moderately effective
☐Not very effective ☐Not effective

37 - G - If yes, did you get yourself into quarantine voluntarily when you had the Covid-19? (including from your family, friends and colleagues) (One answer only)
☐Yes ☐No
☐I don't know/ I don't remember

37 - G1 - If yes, for how many days? (Only one answer)

____

☐Just the time for symptoms.
☐I don't know/I don't remember

37 - G2 - If not, why not? (One or more answers)
☐I didn't know I had to get myself in quarantine
☐I couldn't stop working
☐I couldn't isolate myself from my family.
☐I don't think it is usefull
☐Other: ____

37 - H - If yes, do you think you have any sequels from this disease? (One or more answers)
☐Fatigue ☐Coughing ☐ Shortness of breath ☐Chest pain ☐Headache
☐Smell disorder ☐Taste disorder
☐Muscle pain
☐Other: ____
☐No

37 - I - If yes, do you think you have been sick from Covid-19 a second time since the beginning of the pandemic? (Only one answer)
☐Yes, I did a nasal test and it was positive.
☐Yes, I did a blood test and it was positive.
☐Yes, I had the symptoms of Covid-19.
☐No
☐I don't know, I can't remember

38 - Did you have relatives affected by Covid-19? (One or more answers)
☐Yes, members of my family
☐Yes, friends
☐Yes, colleagues at the gold mining site
☐No
☐I don't know.
☐Other: ____

39 - Did you have any relatives who died from Covid-19? (One or more answers)
☐Yes, members of my family
☐Yes, friends
☐Yes, colleagues at the gold mining site
☐Oui autre :____
☐No
☐I don't know.

40 - How often do you talk about Covid-19 with your relatives? (Only one answer)

☐Never ☐Rarely ☐Sometimes ☐Often ☐All the time

E - Attitudes on vaccination against Covid-19

41 - Are you in favour of vaccination in general? (Only one answer)
☐Very favourable ☐Somewhat favorable ☐Not very favourable
☐Not at all favorable
☐Don't know
☐Other: ____

42 - Do you trust the information provided by the health authorities concerning vaccination in general? (Only one answer)
☐Yes, quite ☐Rather yes
☐No, rather not ☐No, not at all
☐Don't know
☐Other: ____

43 - Do you think that vaccination against Covid-19 is effective? (Only one answer)
☐Yes, quite ☐ Rather yes
☐No, rather not ☐No, not at all
☐Don't know
☐Other: ____

44 - Do you think that vaccination against Covid-19 is safe? (Only one answer)
☐Yes, quite ☐ Rather yes
☐No, rather not ☐No, not at all
☐Don't know
☐Other: ____

45 - Have you ever been vaccinated against Covid-19? (Only one answer)
☐Yes ☐No

45 - A - If yes, where were you vaccinated? (One or more answers)
☐Brazil ☐Guyana ☐Suriname ☐French Guiana
☐Other: ____

45 - B - If yes, how did you get vaccinated? (One or more answers)
☐In a hospital or health center

☐During a vaccination campaign in a town or village
☐During a vaccination campaign on the gold mining site
☐Other: ____
☐No

45 - C - If yes, do you know which vaccine you received? (One or more answers)
☐Pfizer ☐Moderna ☐Astrazeneca ☐CoronaVac ☐Sinopharm
☐I don't know / I don't remember
☐Other:
____

45 - D - If yes, how many doses did you receive? (Only one answer)
☐1 dose ☐2 doses ☐3 doses
☐I don't know / I don't remember
☐Other: ____

45 - E - Where do you plan to take your next dose of vaccine? (Only one answer)
☐In a hospital or health center in France
☐In a hospital or health center in Suriname
☐In a hospital or health center in Brazil
☐During an upcoming vaccination campaign in a town or village
☐Other: ____
☐I don't know.
☐I do not wish to receive an additional injection of vaccine

45 – E1 - If you do not want to receive a new dose of vaccine, why ? (One or more answer)
☐ I think the epidemic is over
☐ I think I am already sufficiently protected against Covid-19
☐ I had serious side effects after the first vaccine dose.
☐Other: ____

45 - F - If not, why did you not get vaccinated against Covid-19? (One or more answers)
☐Not enough informations about vaccination
☐No possibility of vaccination where I am
☐Not available for my situation and age range.
☐No social security
☐No access to the vaccination centre
☐I don't think it's safe
☐I don't think it works.
☐Other:
____

46 - Do you trust Brazilian authorities in the management of Covid-19 health crisis ? (Only one answer)
☐Yes, quite ☐Rather yes
☐No, rather not ☐No, not at all
☐Don't know
☐Other: ____

47 - Do you trust French authorities for the management of Covid-19 health crisis ? (Only one answer)
☐Yes, quite ☐Rather yes
☐No, rather not ☐No, not at all
☐Don't know
☐Other: ____

48 - Do you trust Surinamese authorities to manage the Covid-19 health crisis ? (Only one answer)
☐Yes, quite a bit ☐Rather yes
☐No, not really ☐No, not at all
☐Don't know
☐Other: ____

Is there anything else you would like to add? (Free text)

____
____
____
____
____
____
____
